# Improving Well-Being and Fostering Health-Oriented Leadership among Leaders in Small and Medium-Sized Enterprises (SMEs): A Systematic Review

**DOI:** 10.3390/healthcare12040486

**Published:** 2024-02-17

**Authors:** Rebecca Erschens, Sophia Helen Adam, Carla Schröpel, Mathias Diebig, Monika A. Rieger, Harald Gündel, Stephan Zipfel, Florian Junne

**Affiliations:** 1 Department of Psychosomatic Medicine and Psychotherapy, University Hospital Tuebingen, University of Tuebingen, Osianderstr. 5, 72076 Tuebingen, Baden-Wuerttemberg, Germany; sophia.adam@med.uni-tuebingen.de (S.H.A.); carla.schroepel@med.uni-tuebingen.de (C.S.); stephan.zipfel@med.uni-tuebingen.de (S.Z.); florian.junne@med.ovgu.de (F.J.); 2 Institute of Occupational, Social and Environmental Medicine, Centre for Health and Society, Faculty of Medicine, Heinrich-Heine-University Duesseldorf, Moorenstraße 5, 40225 Duesseldorf, Nordrhein-Westfalen, Germany; mathias.diebig@hhu.de; 3 Institute of Occupational and Social Medicine and Health Services Research, University Hospital Tübingen, University of Tuebingen, Wilhelmstraße 27, 72074 Tuebingen, Baden-Wuerttemberg, Germany; monika.rieger@med.uni-tuebingen.de; 4 Department of Psychosomatic Medicine and Psychotherapy, University Hospital Ulm, Albert-Einstein-Allee 23, 89081 Ulm, Saxony-Anhalt, Germany; harald.guendel@uniklinik-ulm.de; 5 Department of Psychosomatic Medicine and Psychotherapy, Otto von Guericke University Magdeburg, University Hospital Magdeburg, Leipziger Straße 44, 39120 Magdeburg, Saxony-Anhalt, Germany

**Keywords:** intervention, leadership, occupational well-being, small and medium-sized enterprises, stress management

## Abstract

Leaders of small and medium-sized enterprises (SMEs) are often confronted with specific burdens, which frequently result in increased levels of stress. Leadership behaviour, in turn, has a significant impact on employees’ health and performance. Using the Population, Intervention, Comparison, Outcome (PICO) method, we conducted a systematic literature search covering publications from 2002 to 2023 using PubMed, PsycInfo and Business Source Premier on stress-reducing and well-being-improving interventions for SME leaders. The Effective Public Health Practice Project (EPHPP) Quality Assessment Tool was used to assess the methodological quality and risk of bias of the included studies regarding selection bias, study design, confounders, blinding, data collection, withdrawal and drop-out. Of the 3150 identified publications, 6 were included after screening. The studies varied in content (cognitive behavioural therapy [CBT]-based, psychoeducation, and mixed interventions) and approach (individual- and organisation-centred). Not all of the examined interventions provided significant outcomes. However, CBT-based and individualised approaches showed a positive trend in reducing SME leaders’ psychosocial stress and improving their well-being. Despite the limited data, it can be concluded that such interventions are beneficial for leaders and their specific needs. Future research should focus on tailored approaches, derived from well-founded theories and integrative interventions addressing SME leaders.

## 1. Introduction

### 1.1. Work-Related Stress and Strain and Their Consequences

A multidimensional biopsychosocial approach characterises the modern understanding of health. This considers mental health to be context-specific and dependent on a person’s social frame of reference, for instance, the workplace [1,2]. Specific work-related and private stressors, as well as a mismatch of coping strategies and resilience factors, influence the development and maintenance of physical and mental disorders [3]. According to Siegrist [4,5] mental health itself is multifactorial and influenced by biological as well as psychological and social factors. Some of these factors can be found in the work environment [6,7]. They can manifest in a positive sense, for example, through the experience of recognition, the opportunity to be creative and productive or through personal development. Psychosocial stress can also manifest in a negative sense, for example, when work stress arises from potential stressors such as qualitative and quantitative demands, negative leadership behaviour and the experience of injustice [8].

### 1.2. The Significant Role of Small and Medium-Sized Enterprises

Small and medium-sized enterprises (SMEs) play a crucial role in the international labour market, as they constitute the majority of enterprises in industrialised countries. Internationally, SMEs are the most common type of enterprise and the largest group of employers. SMEs account for 99% of enterprises across the EU [9] and 99.4% in Germany, with the majority of all employees in Germany (55.1%) working in an SME [10]. The same applies to Australia (99.8% [11]) and the US, where SMEs account for 50.2% of private sector employment [12]. Thus, SMEs have a major impact on international financial well-being and constitute the majority (>90%) of the non-business economy, employing over 60% of the workforce. Although the definition of an SME is based on the size of the enterprise, the use of the term varies internationally. According to the European Commission guidelines [9], companies with up to 250 employees are most likely to be classified as SMEs, while in the United States, an SME is defined as a company with less than 500 employees [13]. For a global overview, see Gonzales et al. [14].

### 1.3. Small and Medium-Sized Enterprises: Challenges and Opportunities

In their integrative review, Schreibauer et al. [15] described the broad spectrum of psychosocial factors that have been investigated with a focus on the situation in SMEs and concluded that most studies have addressed the aspects of ‘work organisation’ and ‘work content and task’. As demonstrated during the COVID-19 pandemic, SMEs appear to be more vulnerable than larger firms to the antecedents and consequences of financial and economic crises and ultimately to the psychological distress of leaders and followers. Thus, a heavy, multifaceted burden rests on the shoulders of SME leaders as they are exposed to particular demands and stresses [16]. In contrast to large companies, the personalities, skills, attitudes and behaviours of SME leaders are much more in the spotlight, significantly impacting the growth and success of the organisation. SME leaders therefore have a high level of responsibility for the financial security of their employees and the wider social environment, such as their own and their members’ families, as all of these factors are largely dependent on the entrepreneurial decisions of the leader [17]. Due to particular challenges such as business difficulties and the possibility of failure, leaders also have a responsibility towards the well-being of their members in terms of family and personal relationships, financial situation and future career opportunities [18].

### 1.4. Psychological Demands among SME Leaders

Existing research has identified the specific multifaceted challenges of SMEs and their leaders, such as the inability to delegate tasks, pressure of deadlines, high workload, performing tasks outside business hours, being constantly available by e-mail and phone, psychological pressure, job complexity, work–home interference, managing staff, dealing with difficult workplace and staffing issues, lack of appreciation and taking on other tasks at short notice, all of which results in role stress [16,18]. With regard to leaders in SMEs, Wagner et al. [19] described a broad range of challenges as possible hindrances to implementing comprehensive measures to reduce psychosocial stress in their teams, personal challenges (e.g., tasks related to leadership itself, work overload and possible knowledge gaps among managers and owners), general challenges (e.g., demographic change and age-appropriate work design), organisational issues (e.g., vacation planning, sick leave and cross-industry competition for skilled workers), challenges related to team care and staff management (e.g., finding and retaining staff) and challenges regarding the implementation of changes. However, it remains unclear to what extent this specific situation contributes to the increased risk of depressive and anxiety episodes in SMEs relative to larger enterprises [20]. Furthermore, Fernet et al. [21] found an association between feelings of professional loneliness and a higher risk of burnout. Some factors have been identified as having an incremental impact on the mental health of leaders [22] such as workplace and personal relationships and job characteristics like control over working hours.

Cocker et al. [23] also suggested that SMEs are particularly susceptible to emotional contagion due to their small size, specific organisational structure and close contact between leaders and followers, which is based on the assumption that the emotions and behaviours of one person influence the emotions and behaviours of others, a dynamic that is particularly pronounced in everyday moods in work groups [24,25,26]. Despite the apparently high mental health demands in SMEs, surveys from 2011 and 2015 in several EU countries showed that only about 38% of SMEs had carried out risk assessments and only 6% had assessed psychosocial risks [27].

### 1.5. Addressing Mental Health of SME Leaders

Due to the special human and financial resources of SMEs, reduced productivity can be more difficult to deal with, which has a long-term impact on the development and growth of the company [23]. However, the mental health of SME leaders is a neglected area in occupational and psychosomatic health research and practice, despite their contribution to developed economies worldwide.

Mental ill health in particular often leads to long periods of incapacity to work, which is a burden not only on the company and its employees but on the economy as well. There is therefore an urgent need to prevent stress and subsequent health problems in the workplace. Hence, the overall aim of interventions in SMEs is to improve the health of the largest possible proportion of the population by creating health-promoting working conditions and strengthening health literacy in the workplace. Besides comprehensive interventions to reduce psychosocial risk factors at work, workplace training can lead to changes in mindfulness, stress, mental health, well-being and performance at work. In addition, stress management interventions can reduce the risk of stress-related illnesses by promoting sensitivity and practical self-care skills or “resilience”. Interventions can also focus on employee-centred leadership, taking into account individual needs, working conditions and organisational ambitions. Interventions to promote mental health in the workplace through stress management interventions, particularly using cognitive behavioural therapy (CBT) methods, have been shown to be particularly successful [28].

Therefore, interventions to promote stress management in the workplace have been recommended and are often effective in the short term [29,30]. Although prevention programmes to reduce stress at work have shown very good results, their uptake in SMEs has been low and is mostly reserved for larger companies [31,32,33].

### 1.6. Research Aim

Building upon the previously mentioned specific risks, needs, and opportunities for SME leaders, the aim of this systematic review is to provide a comprehensive summary of current research on interventions to improve well-being and promote health-oriented leadership among managers in SMEs. The objectives of this review were to provide an overview of (i) the available interventions targeting the well-being of SME leaders, (ii) the aims and content of the interventions and (iii) the effectiveness of the interventions. Overall, the aim of this review is to contribute to the development of evidence-based strategies to support the health and well-being of SME leaders.

## 2. Methods

### 2.1. General Methodology and Selection Criteria

The procedures and results of this systematic review are presented according to the current Preferred Reporting Items for Systematic Reviews and Meta-Analyses (PRISMA) guidelines [34,35]. The PRISMA checklist for this article can be found in the supplementary materials (Table S1). This review was pre-registered with PROSPERO (CRD42023404710).

#### PICO Criteria

The Population, Intervention, Comparison, Outcome (PICO) [36] scheme within the PRISMA guidelines was used to define the inclusion and exclusion criteria of studies for this review.

Concerning the participants, leaders of SMEs with experience managing more than one follower were included. In line with the internationally varying definitions of SMEs previously mentioned, enterprises with up to 500 employees were included, as well as enterprises that the authors themselves identified as SMEs. Due to the specific environmental factors associated with the health profession, hospitals and health-associated workplaces were excluded, as well as retired leaders and public sectors.

Interventions primarily affecting leaders’ outcomes were included. If applicable, interventions indirectly affecting followers’ outcomes or targeting the leader–follower dyad were included as secondarily relevant. The following intervention aspects were considered: stress management, stress reduction, reduction in burnout, anxiety and depressive symptoms, improvement of quality of life, leader–follower relationship (LMX), transformational/transactional leadership style and improvement of health behaviour (e.g., smoking reduction, healthy eating). Regarding the type of interventions, face-to-face and web-based interventions were included, as well as interventions of all intensities and durations. Interventions targeting solely followers and interventions without a coach/instructor (e.g., apps, self-study or webinar) were excluded.

Regarding the comparator, the presence of a control group was not mandatory.

Concerning the outcomes, subjectively assessed psychosocial outcomes such as distress, anxiety, depression, resilience, coping, an effort–reward imbalance and a leader–follower relationship were included. As current life science and psychophysiology research developments use psychophysiological data in the accompanying research, objectively assessed psychophysiological outcomes such as heart rate variability, cortisol, alpha-amylase and other measures (e.g., number of sick days, resignations) were included. Studies not measuring any health-related outcomes were excluded.

Table 1 provides an overview of the inclusion and exclusion criteria applied. In addition, only peer-reviewed studies published within the last 20 years (2002–2023) in English or German were included in this review. Randomised controlled trials (RCTs) and controlled before–after studies (CBAs) with pre- and post-intervention measurements were included. Qualitative studies and studies with only one measurement point were excluded.

### 2.2. Data Sources and Search Strategy

We conducted a systematic literature search using PubMed, PsycInfo and Business Source Premier until 31 October 2023. The following search terms with Medical Subject Heading (MeSH) terms and combinations were used to search for the relevant literature:

(SME) OR (small and medium-sized enterprise) OR (industry) OR (micro enterprise) OR (small and medium-sized businesses) OR (small business) OR (small company) OR (small enterprise) OR (medium-sized company) OR (medium-sized business) OR (medium-sized enterprise) OR (family business) AND (leader*) OR (manager*) OR (employer) OR (leadership) AND (training*) OR (prevention) OR (program) OR (leadership training) AND (well-being) OR (psychological health) OR (mental health) OR (psychological strain) OR (mental strain) OR (stress reduction) OR (stress prevention) OR (distress) OR (anxiety) OR (anxious) OR (depress*) OR (effort-reward-imbalance) OR (psychophysiol*) OR (cortisol) OR (occupational stress*) OR (employee*) OR (staff) OR (follower*) OR (subordinat*) OR (mental*) OR (psychol*).

To identify further relevant studies, references in the included studies and previous systematic reviews on similar topics were searched for publications using a snowballing technique. Google Scholar, the scientific platform ResearchGate and a manual search on ResearchRabbit.ai were also used to identify additional studies.

### 2.3. Screening Procedure and Data Extraction

Screening was a multistage process involving two reviewers [CS and SHA]. In the first step, the reviewers independently searched the different databases for potential trials and determined the eligibility of a trial based on its title and abstract. Studies that did not meet the criteria were excluded. In the second step, the remaining full-text articles were analysed and assessed separately by the two reviewers according to the inclusion and exclusion criteria.

Subsequently, similarities and divergences were compared. For disagreements between reviewers at any stage of the screening process, an agreement was reached in coordination meetings with senior supervisors [RE and FJ].

Data were extracted from each study by CS and entered into a predefined data extraction table. The extracted data were reviewed and validated by the first authors [RE and SHA]. Information was extracted on location/country, company size and participants, study design, intervention type, delivery mode (e.g., online, face-to-face, telephone), control group, outcome measures, primary outcome (effects on leaders, effects on followers), secondary outcomes and response rates.

### 2.4. Quality Assessment

To assess the risk of bias of the included studies, the Effective Public Health Practice Project (EPHPP) Quality Assessment Tool [37] was used. This tool was chosen because it allows all types of quantitative studies to be assessed and compared and therefore does not require two separate assessment tools for RCTs and non-controlled studies. Considering the common limitations of risk of bias tools, the EPHPP provides comparable accuracy to other commonly used instruments [38].

Bias in the included trials was assessed in eight different domains that could cause possible bias (selection of the population, study design, confounders, blinding, data collection methods, withdrawal and drop-outs, intervention integrity, analysis appropriate to research question). An assessment of the risk of bias was carried out within two parallel, independent processes by two reviewers [SHA and CS]. After each individual decision had been made, the respective ratings were compared again and agreements found. In the case of divergences, the result was discussed until a decision was reached. Disagreements between reviewers were resolved by consensus. Based on the ratings of each area, the final overall assessment of both reviewers was categorized as ‘strong’, ‘moderate’ or ‘weak’.

## 3. Results

### 3.1. Study Selection

The initial systematic search of the databases yielded 3150 results. After removing duplicates, 2879 results remained. After title and abstract screening, 33 studies were deemed eligible. In the full-text screening stage, the most common reason for exclusion (*n* = 13) was a lack of focus on psychosocial outcomes (e.g., occupational safety interventions or trainings focusing on productivity enhancement). Other reasons were that the company size did not meet the inclusion requirements (*n* = 8) as the companies were mostly too large or the trial was conducted within a hospital. Five studies were excluded due to the wrong study design (entirely qualitative interviews or cross-sectional). In addition, two studies were excluded because the interventions did not target leaders but followers, and two other studies were study protocols.

**Figure 1:**
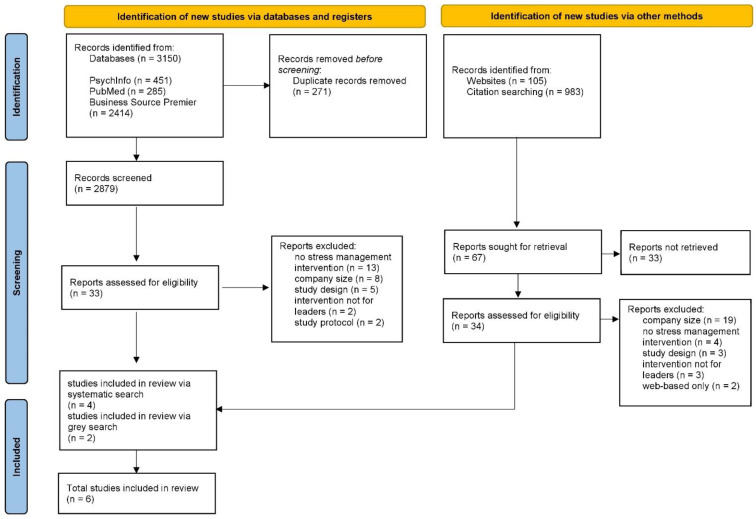
PRISMA flowchart of the systematic literature search.

An additional unsystematic search using citation searches and additional websites (Google Scholar, ResearchGate and ResearchRabbit) yielded a further 1088 results, of which 34 were assessed for eligibility. Once again, in the full-text stage, the size of the companies was found to be a major reason for exclusion (*n* = 19) as they were mostly too large. Also, four of the samples were not found to include a stress management intervention as some studies focused on sale increases and economic success. Moreover, the design of the studies (*n* = 3; e.g., qualitative data only) and the wrong target group (*n* = 3; e.g., the intervention was not explicitly for leaders but was provided to all employees) were identified as reasons for exclusion. The study selection process and reasons for exclusion are shown in the flowchart in Figure 1.

In addition, in the case of uncertainty within the identified studies, authors were contacted by e-mail and asked for clarification. One author was contacted because the size of the company was not clear from the text; this study was subsequently excluded due to its lack of fit. Ultimately, six studies met the applied criteria and were included.

### 3.2. Study Characteristics

Due to the strong heterogeneity of the studies, we have decided against an aggregated presentation of the studies in this article. Some core characteristics are listed below; for an aggregated overview on a quantitative level, see Table 2. If specified in the studies, the size of the SMEs ranged from less than 20 employees [39] to less than 500 employees [40]. Also, the occupational sectors were found to be heterogeneous across and within the studies, varying from a sake brewery [41] to the social assistance sector [40] to small craft businesses (e.g., hairdresser or painter [42]), as well as mixed samples in Saraf et al. [43] (service sector, manufacturing and retailing), Martin et al. [44]( transport, finance and retail) and Schwatka et al. [45]. The gender distribution showed to be either more male or more female. A higher ratio of men was found in Saraf et al. [43] (95.7%), Takao et al. [41] and Busch et al. [42](mostly male). A higher proportion of women was found in Schwatka et al. [40] (74%), Martin et al. [44] (57–71%, depending on the group) and Hansen et al. [39] (mostly female, although the information was only available for one intervention group). A detailed overview of the study characteristics and the samples is provided in Table 3. It is important to note that inconsistent terminology for ‘leader’ (e.g., ‘SME manager’, ‘SME owner’) was used across the included papers.

### 3.3. Detailed Description of Included Study Results

Since our search only included six studies and the studies show great heterogeneity in terms of content and concept, the following sections go into detail about the individual studies. Using an iterative process among the involved authors, we decided to cluster the included interventions as follows:

1. *Leadership- and well-being-oriented interventions*
2. *Cognitive behavioural theory (CBT)-based and goal-oriented interventions*
3. *Individualised coaching-based interventions*

#### 3.3.1. Well-Being- and Leadership-Oriented Interventions

Hansen et al. [39] conducted a pre-post study in Norway and Sweden and implemented a leader-based workplace health intervention to support the psychosocial and health-related environment of SMEs. Within a four-phased model over the course of one year, two sub-interventions were tested (the Norwegian and Swedish intervention models) to identify regional differences. In the first phase of the Norwegian intervention model, company visits, questionnaires and interviews, as well as a physical fitness test with SME leaders, were carried out to investigate working and health conditions among SME leaders. The second phase focused on the implementation of a counsellor-led intervention called “Leadership in Modern Working Life”. The intervention included three meetings over the course of a year where leaders discussed several topics like team development, handling conflicts and work pressure. In the third phase, the leaders received individualised support from the counsellors on leadership behaviour and other relevant issues. The fourth phase was mainly composed of follow-up measures analogous to the first phase. The Swedish model was designed in the same way as the Norwegian model, except that the first phase also involved a diagnostic interview with a psychologist or nurse to assess working conditions and leadership. Another difference was that leaders received eight meetings over the course of a year. Also, the fourth phase included an examination and a discussion with an occupational health nurse. The authors carried out a holistic assessment of health using the Salutogenic Health Indicator Scale (SHIS; [56]), which includes the domains of perceived stress, illness, energy level, physical function, happiness level, psychosomatic function, emotional expression, and cognitive and social functioning. For outcomes at the organisational level, the Nordic Questionnaire on Positive Organisational Psychology (N-POP; [52]) and the Work Experience Measurement Scale (WEMS; [57]) were used. Compared to a reference group of SMEs that did not receive any intervention, the intervention groups only showed marginal effects regarding psychosocial working conditions and health outcomes. However, the authors could show significant intervention effects regarding the secondary outcomes of external job performance and sickness absences.

Schwatka et al. [40] conducted an RCT and assessed the effectiveness of the Total Worker Health (TWH) leadership development programme in the United States. TWH was compared to a control group that received an already established intervention (Health Links; Tenney et al., [65]) that included an online health assessment on health at the workplace, plus additional counselling. The training was not only provided to senior SME leaders but also to one additional staff member per SME, mostly human resources or safety managers. In addition to the Health Links intervention, over the course of a year, the intervention group also completed the TWH leadership development programme, which was grounded in leadership theories and provided in a blended format (online and face-to-face). During an in-person session of six hours, achievable goals were developed, including in the areas of safe and healthy workplaces, being a role model and facilitating organisational change. Within three months after training, participants received further training modules consisting of up to three 30 min coaching sessions and an online social goal tracking platform. They assessed employee-reported healthy leadership practices, including leader communication, role modelling, employee recognition, resource allocation and accountability by using Lee et al.’s six-item organisational commitment to safety scale (CTSS; [48]. They also assessed the safety and health climate of the organisation using a health climate scale (HCS; [49]). The authors were unable to find any significant group differences regarding well-being, health behaviour or any other outcome variable.

Takao et al. [41] conducted an RCT in Japan. They focused on a different topic, investigating the provision of support to leaders in a tense working environment by comparing a waitlist control group to an intervention group that received a 60 min, single-session educational programme on the role of supervisors in mental health awareness, self-care and coping with mental illness in the workplace. Moreover, the study implemented role-play exercises for active listening provided by two psychologists. It is noteworthy that the aim of this study was to obtain outcome measures only for the followers, using a questionnaire derived from the Brief Job Stress Questionnaire [47]. However, the recipients of the intervention were the leaders. No significant group differences were found in the reduction in psychological distress, but trends towards a reduction in psychological distress were found in several sub-populations, as the intervention showed to be specifically beneficial for male, white-collar followers under the age of 34 years. However, psychological distress among women did not change for either group, but an upward trend for job performance was shown among the intervention group.

#### 3.3.2. CBT-Based and Goal-Oriented Interventions

In the RCT by Saraf et al. [43], the authors tested a five-week, group-based CBT intervention called Problem Management Plus for Entrepreneurs (PM+E) in a region affected by “fragility, conflict, and violence” in Pakistan. Recruited via a cash grants programme, all included SME leaders in both groups received about USD 14,300 to rebuild or establish a business in Pakistan. Moreover, the intervention consisted of CBT-based content like stress and problem management strategies, behavioural activation and the improvement of social support by utilizing stress-inducing anonymised real-life case studies and scenarios. The sessions, each lasting about three hours, were provided face-to-face and led by trained specialists. By using the Patient Health Questionnaire Anxiety and Depression Scale (PHQ-ADS; [54]) and the WHO-5 Wellbeing Index [58], the authors were able to find significant reductions in the treatment group in terms of depression and anxiety symptoms and prevalence rates, as well as a significant increase in well-being compared to the control group. For the sub-population of those having mild to moderate levels of depression and anxiety, the effects were found to be particularly accentuated.

Martin et al. [44] conducted an RCT in Australia and analysed two types of mental health promotion interventions (self-administered and telephone-supported) compared to an active waitlist control group. The self-administered intervention consisted of a 60 min DVD programme covering relevant CBT-based topics for mental health among SMEs such as the use of coping mechanisms and positive relationships using real-life case studies of SME leaders. Participants were also provided a manual including information on depression, anxiety, workplace stress and bullying. The self-administered intervention plus telephone support included six 30 min telephone calls with a psychologist to assist in the implementation of the content taught in the DVD. The active control group received a minimal content intervention using parts of the manual and the DVD. Regarding measurement instruments, the Kessler K-10 Screening Scale for Psychological Distress [50] was used. The authors found a greater ratio of change in psychological distress in the active control group and in the self-administered plus telephone group but not in the self-administered only group.

#### 3.3.3. Individualised Coaching-Based Intervention

Another type of intervention was investigated by Busch et al. [42] based on the assumption that spouses may have a strong influence on the psychological well-being of SME leaders and help them to better distance themselves from work and alleviate stress. Derived from the theoretical framework of the Zurich Resource Model (ZRM [60]) and the Rubicon phase model of change [59], Busch et al. analysed the effects of a blended couple coaching intervention [66] compared to a waitlist control group using a German sample. Employing a quasi-experimental design, over the course of four months, five coaching sessions including face-to-face meetings and online sessions were conducted. The authors used a validated German version of the Maslach Burnout Inventory [51], the validated German version of the Positive and Negative Affect Scale (PANAS; [53]), and items from the Recovery Experience Questionnaire (REQ; [55]) to measure psychological detachment from work. Among other topics, the content of the sessions was self-reflection and health-related goal setting. The authors only found significant changes in the decrease in emotional exhaustion and a significant effect with regard to detachment within the intervention group compared to the control group.

### 3.4. Risk of Bias in the Included Studies

**Figure 2:**
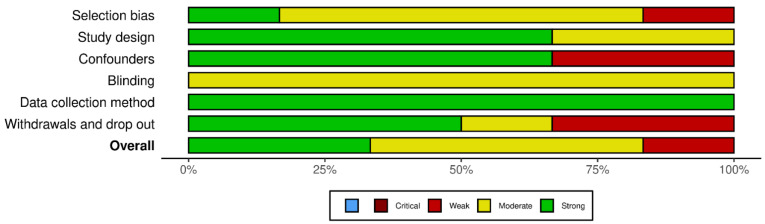
Visualized risk of bias assessment summary for each included study indicating the overall quality of each domain in accordance with the guidelines of the EPHPP Quality Assessment Tool [37].

The overall quality of the studies was rated as strong in two articles [41,43], indicating a low risk of bias (no weak rating in any area). Three articles [39,40,44] received an overall rating of ‘moderate’ (one weak rating), and one article [42] received an overall rating of ‘weak’ (two or more weak ratings). Figure 2 shows the overall percentage of studies with a high, moderate or low risk of bias for each of the criteria using the robvis visualisation tool [67]. Moderate or weak ratings mainly resulted from the fact that blindness was not adequately explained or not possible or due to the nature of the interventions.

**Figure 3:**
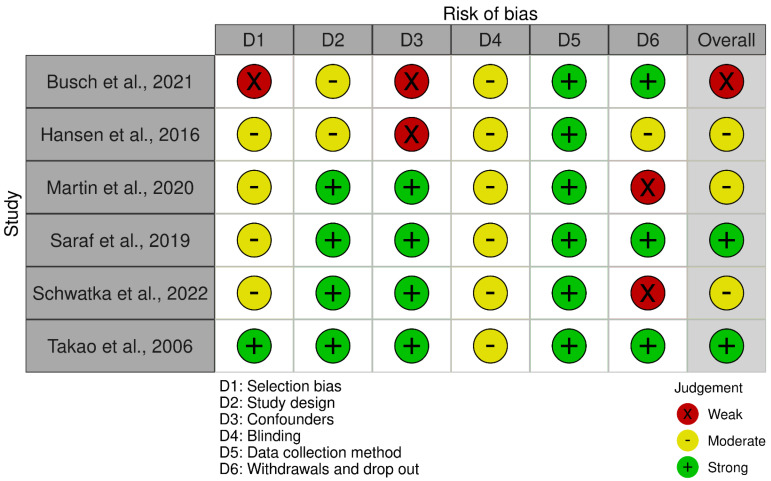
Visualized risk of bias assessment for each included study [39,40,41,42,43,44] and each domain indicating the assessor’s judgements in accordance with the guidelines of the EPHPP Quality Assessment Tool [37]. Please note that when using a different rating tool, the overall and domain-specific assessment may vary.

Moreover, the lack of reporting of a response rate also led to a deduction of the quality rating. It is particularly noteworthy that the domain “data collection method” was consistently rated as “strong”. This is due to the fact that a qualitatively high standard had already been set for the outcome measures by applying the PICO criteria. A plot of the domain-level judgements for each individual result is provided in Figure 3. A detailed overview of the ratings can be found in Table 4. It is important to emphasize that the assessment is based on the Effective Public Health Practice Project (EPHPP) Quality Assessment Tool [37], selected for this review, and its associated categories and rating options. When using a different rating tool, the overall and domain-specific assessment may vary.

## 4. Discussion

Small and medium-sized enterprises (SMEs) play an essential role in economic development, and their leaders often face unique challenges in managing their own health and well-being alongside the demands of running their businesses. However, supportive interventions for SME leaders remain an under-researched area. To the best of our knowledge, this is the first systematic review of the literature with a primary focus on interventions to promote the health of SME leaders. The synthesized results show that CBT-based and individualised approaches are especially helpful in reducing the psychosocial stress factors of SME leaders and improving their well-being.

In the synopsis of the studies, it became clear that the six studies included are very heterogeneous in terms of content and that a direct comparison of the content is therefore not entirely appropriate in order to assess the benefits of the respective interventions.

Also, as the included studies included vary regarding their methodology, this might also affect the strength of the evidence. Moreover, the clustering suggested above shows the diversity of approaches to support SME leaders. However, among the interventions reviewed, those that directly target leaders’ well-being appear to be more effective than those that indirectly target employees’ well-being. It therefore seems that the interventions only have a marginal effect on the outcome variables for the direct followers. In the same vein, a Cochrane review by Kuehnl et al. [68] found no consistent findings on whether leadership training has an effect on employee well-being.

Our review’s results are also consistent with previous findings that organisation-level interventions are more difficult to implement than individual-level interventions [69]. Also, as can be seen in the studies by Saraf et al. [43] and Takao et al. [41] in particular, environmental factors and cultural influences on the effectiveness of interventions should also be taken into account, as these seem to have a major impact on the effects of health support interventions. During the course of the systematic literature review, it became clear that, despite the existence of quite a large number of interventions for SMEs, very few were specifically designed for leaders or with the particular aim of reducing their stress levels. The studies found in the systematic search often dealt with aspects of occupational safety or interventions that assessed an economic outcome rather than psychological or health-related outcomes. There was also a great deal of heterogeneity in the content and aims of the interventions themselves, including face-to-face, technology-based and blended interventions, as well as an intervention to assess spousal support. In addition, a minority of studies measured employee-related stress variables.

The public health relevance of this issue is high. Workplace stress is a known risk factor for a range of mental and somatic illnesses. Standard health promotion measures for managers are currently insufficiently implemented. Primary interventions in this area can prevent illness and the associated days of incapacity to work, as well as cost-intensive curative and rehabilitative measures. Many employees can be reached, especially in SMEs. The well-being of entrepreneurs is arguably higher compared to other workers [70]. This is attributed to the autonomy and choices entrepreneurs have to meet their psychological needs; they are often self-motivated and enterprising and have clear goals for their business, which can make them more resilient to external pressures [18]. On the other hand, Yeh et al. [71] reported higher job demands, greater job insecurity, less job autonomy and lower career prospects in SMEs compared to large private companies and the public sector. Dawkins et al. [32] identified barriers to implementing stress management interventions in SMEs such as the lack of resources, support and competence to support these programmes. In addition, SME owners/managers are often too busy with the day-to-day issues of running their business, leaving limited or no time to engage in the implementation of health promotion training and skills development [19]. Furthermore, engagement in workplace health promotion programmes decreases with the size of the company, suggesting that small companies in particular have little opportunity to benefit from interventions.

### Strengths and Limitations

Overall, this review makes a relevant contribution to research on current challenges regarding the content, implementation pathways and impact of health promotion and primary prevention in the context of SMEs. This comprehensive literature review was conducted by three researchers [CS, RE and SHA] using systematic tools and grey-search paths, including innovative platforms such as ResearchRabbit.ai. Moreover, we conducted an extensive risk of bias assessment, giving comprehensive insight into the quality of the included studies.

This study has several limitations. First, only six interventions were included. Moreover, the majority of the included studies were assessed as having a medium to high risk of bias. In addition, the high proportion of studies showing positive effects of the investigated interventions suggests that studies without corresponding positive effects were not published; in other word, there may be a publication bias. The results should therefore be interpreted with caution. Also, the participants were homogeneous in terms of age as the populations were mostly middle-aged managers. Therefore, it cannot be excluded that interventions for other age groups might yield different results. Moreover, the industries differed greatly from one another, which may impact the effectiveness of interventions; the retail industry, for example, is subject to different stressors than the construction industry. Future research should therefore explicitly focus on different sub-populations of SME leaders in terms of individual and organisational traits. Due to the heterogeneity of the studies and the complexity of designing these trainings, no pattern could be identified. Therefore, further studies are needed to derive differentiated cluster effects. Furthermore, it should be noted that the population of SME leaders investigated here constitutes a highly specific target group. This could potentially limit the generalizability of the findings to other groups, including other groups of managers, e.g., those of larger companies. Moreover, according to the manual of the applied risk of bias tool, intervention integrity and the conducted analyses were not considered in the overall evaluation, which may lead to an incomplete picture of the quality of the assessed studies.

## 5. Conclusions

A number of possible implications for management practice can be derived from the present synthesized findings and the existing research background on the topic of SME leaders. Firstly, due to the heterogeneity of the participating SMEs and the needs of the leaders, it is necessary to provide individual, personalised offers. These should ideally be close to the needs of those concerned in order to provide them with a tailor-made offer, which is why it is best to carry out a needs assessment beforehand. Drawing conclusions from the summarised findings, there is evidence that more theory-based interventions are needed, preferably based on established group CBT interventions. In addition, as a lack of peer interaction has been identified as a major source of distress [23], interventions could also aim to increase interaction and decrease feelings of isolation and loneliness on a daily basis.

In preparing this systematic review, we also considered a number of challenges associated with studying mental health and adapting interventions for leaders in SMEs:

1. Definition: As mentioned above, the definition of SMEs varies internationally and between organisations, which often makes it difficult to correctly identify types of businesses and their specific challenges [14].
2. Limited research: There is a lack of research specifically focused on the mental health of SME leaders. Most studies on this topic tend to be limited in scope or are conducted on larger organisations, making it difficult to draw generalisations or make informed recommendations for SMEs [72].
3. Sample size and representativeness: SMEs are a diverse group, and their leaders may have unique experiences and needs. However, the small sample sizes in most studies and the lack of diversity in the samples can make it difficult to generalise findings to the wider population of SME leaders.
4. Access and recruitment: SMEs are often more difficult to access for research purposes than larger organisations as they may not have dedicated HR departments or may be more reluctant to participate in research. This can make it difficult to recruit participants, which may further limit the generalisability of the findings [32].
5. Self-reporting bias: Mental health research often relies on self-reported data, which can be subject to biases such as social desirability or recall bias. This can make it difficult to accurately assess the mental health of SME leaders, particularly if they are reluctant to disclose mental health issues [73].
6. Lack of resources and support: SMEs may have limited resources to devote to mental health initiatives, which can make it difficult to support the mental health of leaders and employees. In addition, SME leaders may feel a greater sense of responsibility for the success of their business, which can create additional stress and make it more difficult to prioritise their own mental health [32].

Health-oriented leadership styles are known to yield positive outcomes for both leaders and followers (for an overview, see [8]). Thus, future research on the wellbeing of SME leaders should focus more specifically on health-oriented leadership regarding outcomes and intervention content. We thus recommend considering focused attention on leadership styles like relational leadership (LMX; e.g., [74]), as well as transformational and transactional leadership (e.g., [75,76]) in future studies.

## Figures and Tables

**Table 1:** PICO criteria applied for inclusion of studies.

PICO Criteria	Inclusion	Exclusion
Participants	Leaders of small and medium-sized enterprises (SMEs) Experienced in managing more than one follower	Studies conducted in hospitals or health-care settings Public sectors Retired leaders
Intervention	Interventions with direct impact on leaders’ outcomes Interventions with indirect impact on followers’ outcomes or targeting the leader–follower dyad (if available) Content: Stress management, stress reduction, reduction in anxiety and depressive symptoms, improvement of quality of life, leader–follower relationship (LMX), transformational/transactional leadership style, improvement of health behaviour Type of intervention: face-to-face or web-based/online interventions All intensities or durations	Interventions targeting solely followers Interventions without a coach/instructor
Comparator	Control group not mandatory	
Outcome	Subjectively assessed psychosocial outcomes (e.g., distress, anxiety, depression, resilience, coping, effort–reward imbalance, leader–follower relationship) Objectively assessed psychophysiological outcomes (e.g., heart rate variability, cortisol, alpha-amylase) Other objectively assessed measures (e.g., number of sick days, resignations)	Studies without health-related outcome measures

**Table 2:** Aggregated quantitative summary of the descriptive data.

Studies (*n* = 6)		
Sample size	Total *(n)*	4063
	Minimum sample size *(n)*	32
	Maximum sample size *(n)*	2785
Age	Mean age range *(M)*	41.7–51
Study design	RCT (*n*)	4
	Quasi-experimental (*n*)	1
	Pre-post study (*n*)	1
Instruments	Sub-syndromal	
	*Stress*	BJSQ (*n* = 1) Kessler K-10 (*n* = 1)
	*Well-being*	Five-item scale by Staehr (*n* =1) SHIS (*n* = 1) REQ (*n* =1) WHO-5 (*n* = 1)
	*Burn-out*	MBI (*n* = 1)
	*Affect*	PANAS (*n* = 1)
	Syndromal	
	*Anxiety and depression*	PHQ-ADS (*n* = 1)
	Leadership	
		CTSS (*n* = 1) HCS (*n* = 1) N-POP (*n* = 1) WEMS (*n* =1)
Response rates (%)		37–96
Country—context		
	Australia	(*n* = 1)
	Germany	(*n* = 1)
	Japan	(*n* = 1)
	Norway and Sweden	(*n* = 1)
	Pakistan	(*n* = 1)
	USA	(*n* = 1)

Note. Five-item scale by Staehr [46]; BJSQ: Brief Job Stress Questionnaire [47]; CTSS: commitment to safety scale [48]; HCS: health climate scale [49]; Kessler K-10: Kessler K-10 Screening Scale for Psychological Distress [50]; MBI: German version of the Maslach Burnout Inventory [51]; N-POP: Nordic Questionnaire on Positive Organisational Psychology [52]; PANAS: Validated German version of the Positive and Negative Affect Scale [53]; PHQ-ADS: Patient Health Questionnaire Anxiety and Depression Scale [54]; REQ: Recovery Experience Questionnaire [55]; SHIS: Salutogenic Health Indicator Scale [56]; WEMS: Work Experience Measurement Scale [57]; WHO-5: WHO-5 Well-Being Index [58].

**Table 3:** Overview of the content of the included interventions.

First Author (Year of Publication), Country Implemented	Recipients of the Intervention	Number of Followers per Leader	Outcome Measures Available for (Follower/Leader/Both)	Format	Framework
Busch et al. (2021), Germany [42]	Dyads of small business owners and their respective spouses	Intervention group: on average, 6 followers. Control group: on average 13 followers	Leader	Blended: online and face-to-face	Rubicon phase model [59] Zurich Resource Model [60]
Hansen et al. (2016), Norway and Sweden [39]	SME leaders	<20 followers	Leader and follower	Face-to-face and telephone contacts	European Network for Workplace Health Promotion [61]
Martin et al. (2020), Australia [44]	SME leaders	0–200 followers	Leader	Self-administered and telephone supported	Psychological capital: work-related hope, optimism, resilience, and self-efficacy [62]
Saraf et al. (2019), Pakistan [43]	SME leaders	On average, 10 followers	Leader	Face-to-face	Cognitive behavioural therapy core elements (stress management, problem solving, behavioural activation, strengthening support network, self-care) adapted for leaders working within fragility, conflict, and violence surroundings
Schwatka et al. (2022), United States of America [40]	SME leader and one additional organization member (safety manager or human resource manager)	<500 followers	Follower and leader	In-person and virtual components	Multi-level model of safety [63]
Takao et al. (2006), Japan [41]	SME leaders	On average, 5.5 follower	Follower	Face-to-face	Theoretic background by Theorell et al. [64] assuming that training programs to improve the psychosocial competence of managers reduce stressors among leaders and employees

**Table 4:** Detailed overview of the reasons for the two raters’ risk of bias ratings for each study and each domain.

Busch et al., 2021		
Bias Domain	Authors Judgement	Reasons for Assessment ^1^
Selection bias	Weak	selection not described no response rate described
Study design	Moderate	quasi-experimental
Confounders	Weak	sample was limited to mainly male leaders different sizes of employees per leader in intervention and control group identified as relevant confounder; control for this confounder was not reported
Blinding	Moderate	it is not clear if the outcome assessors were aware of the intervention or status of the participants the participants were aware of the research question
Data collection method	Strong	tools for outcome measures were reliable and valid
Withdrawals and drop-out	Strong	80–100% of the participants completed the study
**Hansen et al., 2016**		
**Bias Domain**	**Authors Judgement**	**Reasons for Assessment**
Selection bias	Moderate	selected individuals were very likely to be representative of the target population no response rate described
Study design	Moderate	cohort analytic (two intervention groups)
Confounders	Weak	control of confounders was not described
Blinding	Moderate	it is not clear if the outcome assessors were aware of the intervention or status of the participants the participants were aware of the research question
Data collection method	Strong	tools for outcome measures were reliable and valid
Withdrawals and drop-out	Moderate	69% of the participants completed the study
**Martin et al., 2020**		
**Bias Domain**	**Authors Judgement**	**Reasons for Assessment**
Selection bias	Moderate	selected individuals were very likely to be representative of the target population no response rate described
Study design	Strong	RCT
Confounders	Strong	no important differences between the groups
Blinding	Moderate	blinding was not described
Data collection method	Strong	tools for outcome measures reliable and valid
Withdrawals and drop-out	Weak	49.5% of the participants completed the study
**Saraf et al., 2019**		
**Bias Domain**	**Authors Judgement**	**Reasons for Assessment**
Selection bias	Moderate	response rate less than 80%
Study design	Strong	RCT
Confounders	Strong	relevant confounders controlled
Blinding	Moderate	blinding is not described
Data collection method	Strong	tools for outcome measures were reliable and valid
Withdrawals and drop-out	Strong	low drop-out rate
**Schwatka et al., 2022**		
**Bias Domain**	**Authors Judgement**	**Reasons for Assessment**
Selection bias	Moderate	selected individuals were very likely to be representative of the target population no response rate described
Study design	Strong	RCT
Confounders	Strong	relevant confounders controlled
Blinding	Moderate	blinding was not described
Data collection method	Strong	tools for outcome measures were reliable and valid
Withdrawals and drop out	Weak	37% of the participants completed the study
**Takao et al., 2006**		
**Bias Domain**	**Authors Judgement**	**Reasons for Assessment**
Selection bias	Strong	Participation rate: 96%
Study design	Strong	RCT
Confounders	Strong	relevant confounders controlled
Blinding	Moderate	blinding was not described
Data collection method	Strong	tools for outcome measures were reliable and valid
Withdrawals and drop-out	Strong	low drop-out rate

^1^ Please note that when using a different rating tool, the overall and domain-specific assessment may vary.

## Data Availability

All of the data are included in this report.

## Supplementary Materials

The following supporting information can be downloaded at: https://www.mdpi.com/article/10.3390/healthcare12040486/s1, Table S1: PRISMA checklist 2020.
